# MicroRNA-218 and microRNA-520a inhibit cell proliferation by downregulating E2F2 in hepatocellular carcinoma

**DOI:** 10.3892/mmr.2015.3516

**Published:** 2015-03-19

**Authors:** YE DONG, JIANJUN ZOU, SAN SU, HUIYI HUANG, YANZHEN DENG, BIRONG WANG, WEIDONG LI

**Affiliations:** 1 The 1st Ward of the Medical Department, Affiliated Cancer Hospital of Guangzhou Medical University, Guangdong 510095, P.R. China; 2 Department of Oncology, Guangzhou Chest Hospital, Guangzhou, Guangdong 510095, P.R. China

**Keywords:** microRNA-218, microRNA-520a, E2F2, hepatocellular carcinoma

## Abstract

Hepatocellular carcinoma (HCC) is the fifth most common cancer type worldwide and the third leading cause of cancer-associated mortality. To date, its pathogenesis has remained poorly understood. Previous studies have demonstrated that deregulated microRNA (miR) participates in hepatocarcinogenesis. In the present study, miR-218 and miR-520a were observed to be downregulated in human HCC cells relative to normal hepatic cells. Overexpression of miR-218 or miR-520a inhibited cell proliferation and induced cell cycle arrest at the G_0_/G_1_ phase checkpoint. Furthermore, a dual-luciferase reporter assay identified that E2F2 was a novel direct target of miR-218 but not miR-520a in HCC. In addition, miR-218 and miR-520a were observed to negatively regulate E2F2 mRNA and protein levels. This suggested that miR-218 regulated the expression of E2F2 via directly binding to its 3′-untranslated region, whereas miR-520a affected E2F2 expression indirectly. In conclusion, these results indicated that miR-218 and miR-520a are crucial in the development of HCC via the inhibition of cell proliferation and cycle progression by downregulating E2F2.

## Introduction

MicroRNAs (miRNAs) are a class of small, endogenously expressed, well-conserved noncoding RNAs with 18–25 nucleotides. These RNA molecules suppress protein expression predominantly by base pairing with the 3′ untranslated region (3′UTR) of their target mRNA (1). miRNAs are considered as powerful post-transcriptional regulators of various biological processes, including cell proliferation, migration, differentiation and apoptosis (2–4). Previous studies have demonstrated that miRNA dysregulation is closely associated with the development and progression of various types of human cancer (5,6).

Hepatocellular carcinoma (HCC) is the fifth most common cancer type worldwide and the third leading cause of cancer-associated mortality, resulting in ~700,000 mortalities each year (7,8). Although its mortality was reduced due to the advancements of liver transplantation and surgical resection, the long-term prognosis remains unsatisfactory due to late-stage diagnosis and the high recurrence rate (9). It is widely accepted that environmental factors and epigengetic/genetic alterations cooperate in the initiation and progression of HCC (10). miRNA expression profiles have demonstrated that a subset of miRNAs were aberrantly expressed in HCC (10,11). Furthermore, several deregulated miRNAs have been validated to regulate HCC cell proliferation, migration and apoptosis (12–16). These observations indicated that the dysregulation of miRNAs may be implicated in the generation and progression of HCC.

Previous studies have demonstrated that miRNA-218 (miR-218) and miR-520a are associated with tumor pathogenesis and the development of various types of human cancer. The expression of miR-218 is downregulated and may serve as a potential tumor suppressor in glioblastoma, clear cell renal cell carcinoma and gastric, nasopharyngeal, cervical, breast, oral and non-small cell lung cancer (17–24). In addition, Li *et al* (25) reported that miR-218 is downregulated in HCC tissues and may inhibit cell proliferation and promote cell apoptosis. Regarding miR-520a, fewer studies have been performed; however, it has been reported to inhibit cell proliferation and invasion by directly targeting ErbB4 in esophageal squamous cell carcinoma (26). However, the role of miR-218 and miR-520a in HCC and the molecular mechanisms by which the miRNAs exert their functions have remained elusive.

In the present study, it was hypothesized that miR-218 and miR-520a are downregulated in HCC cells compared with normal hepatic cells. In addition, the restoration of miR-218 and miR-520a was suggested to inhibit cell proliferation by inducing cell cycle arrest at the G_0_/G_1_ phase checkpoint. The present study aimed to provide evidence that miR-218 directly targets E2F2 to regulate its expression in HCC. Additionally, miR-520a was hypothesized to affect E2F2 expression.

## Materials and methods

### Cell culture and transfection

The human HCC cell lines HepG2, Huh7, MHCC-97H, BEL-7402 and the normal hepatic cell line L02 were obtained from the Type Culture Collection of the Chinese Academy of Sciences (Shanghai, China). The cells were maintained at 37°C under 5% CO_2_ in Dulbecco’s modified Eagle’s medium (Gibco-BRL, Invitrogen Life Technologies, Carlsbad, CA, USA) supplemented with 10% fetal bovine serum (Gibco-BRL). Mimics of miR-218 and miR-520a and the negative control (NC) were purchased from Shanghai GenePharma, Co., Ltd. (Shanghai, China). miRNA transient transfection was conducted with Lipofectamine 2000 (Invitrogen Life Technologies) according to the manufacturer’s instructions.

### RNA extraction and reverse transcription-quantitative polymerase chain reaction (RT-qPCR)

Total RNA from cultured cells was extracted using TRIzol reagent (Invitrogen Life Technologies). In order to measure miR-218 and miR-520a expression levels, cDNA was synthesized using the TaqMan miRNA Reverse Transcription kit (Applied Biosystems, Life Technologies, Thermo Fisher Scientific, Waltham, MA, USA). The expression levels of miR-218, miR-520a and the endogenous control U6 were quantified using the TaqMan MicroRNA Assay kit (Applied Biosystems). To estimate the mRNA levels of E2F2, a total of 500 ng total RNA was reverse-transcribed using the PrimeScript RT reagent kit (Takara Biotechnology Co., Ltd., Dalian, China). RT-qPCR was conducted using the 7500 Real-Time PCR system (Applied Biosystems) using SYBR Premix Ex Taq (Takara Biotechnology Co., Ltd.) and β-actin was used as an internal control. The primers used in the present study were designed and synthesized by GeneCore BioTechnologies Co., Ltd. (Shanghai, China); the sequences were as follows: E2F2 forward, 5′-CGT CCC TGA GTT CCC AAC C-3′ and reverse, 5′-GCG AAG TGT CAT ACC GAG TCT T-3′; and β-actin forward, 5′-AGG CAC CAG GGC GTG AT-3′ and reverse, 5′-TGC TCC CAG TTG GTG ACG AT-3′. Each sample was run in triplicate.

### Western blot analysis

Total proteins were extracted from cells using radioimmunoprecipitation assay lysis buffer (Sigma-Aldrich, St. Louis, MO, USA) and quantified by the Bradford assay (Bio-Rad Laboratories, Inc., Hercules, CA, USA). Equal amounts of protein were separated using 8% SDS-PAGE (Affymetrix, Inc., Santa Clara, CA, USA) prior to being transferred to polyvinylidene difluoride membranes (Bio-Rad Laboratories, Inc.). Following blocking with 5% skimmed milk, the membranes were incubated with rabbit anti-human E2F2 polyclonal antibody (1:200; sc-632; Santa Cruz Biotechnology, Inc., Santa Cruz, CA, USA) overnight at 4°C. Subsequent to washing with Tris-buffered saline (Affymetrix, Inc.) containing Tween 20 (TBST; Sigma-Aldrich), horseradish peroxidase-conjugated secondary goat anti-rabbit immunoglobulin G antibodies (1:1,000; Santa Cruz Biotechnology, Inc.) were incubated with membranes for 1 h at room temperature. Following washing again using TBST, the protein bands were detected by chemiluminescence (Amersham ECL Plus Western Blotting Detection system; GE Healthcare Life Sciences, Pittsburgh, PA, USA). Digital images were captured using a chemiluminescent imaging system (FluorChem SP; Alpha Innotech, San Leandro, CA, USA). β-Actin was used as a protein-loading control. The intensity of the protein fragments was quantified using Quantity One software, version 4.5.0 (Bio-Rad Laboratories, Inc.).

### Cell proliferation and colony formation assays

Cell proliferation was determined using MTT assays. Following transfection with NC, miR-218 or miR-520a, respectively, for 48 h, Huh7 and MHCC-97H cells were plated in 96-well plates at a density of 3,000 cells/well. Following the plating of the cells for 12, 24, 36 and 48 h, 20 *μ*l MTT (Sigma-Aldrich) was added to each well. Following incubation with MTT for an additional 4 h at 37°C, the cells were lysed in 150 *μ*l dimethyl sulfoxide (Sigma-Aldrich) and cell proliferation was evaluated by measuring the absorbance at 490 nm (SpectraMax M5; Molecular Devices, Sunnyvale, CA, USA). For the colony formation assays, 400 cells were plated onto six-well plates and incubated at 37°C until the cells grew to visible colonies. The colonies were washed with phosphate-buffered saline (PBS) twice, fixed with methanol (Sigma-Aldrich) and stained with 0.1% crystal violet (Sigma-Aldrich), then the numbers of colonies per well were counted. All assays were performed in triplicate.

### Cell cycle analysis

Huh7 and MHCC-97H cells were plated in 60-mm dishes and transfected with NC, miR-218 or miR-520a, respectively. At 48 h post-transfection, the cells were harvested and washed with PBS, then fixed in 70% ethanol overnight at 4°C. Subsequent to washing in cold PBS three times, the cells were incubated with 100 *μ*l RNase (Nanjing KeyGen Biotech. Co. Ltd., Nanjing, China) for 30 min at 37°C and were stained with 400 *μ*l propidium iodide (Nanjing KeyGen Biotech. Co. Ltd.) for an additional 30 min. Samples were then analyzed for the cell-cycle distribution using a BD FACSCalibur Cell Analyzer (BD Biosciences, Franklin Lakes, NJ, USA).

### Dual-luciferase reporter assay

Target genes of miR-218 and miR-520a were assessed using the miRNA target prediction tool miRanda (http://www.microrna.org). To investigate whether E2F2 is a direct downstream target gene of miR-218 and miR-520a, dual-luciferase reporter assays were performed. The wild-type (WT) 3′-UTR of E2F2 containing the potential binding site of miR-218 or miR-520a and the corresponding mutational 3′-UTR of E2F2 were cloned into the psiCheck2 dual luciferase reporter vector (Promega Corporation, Madison, WI, USA). Huh7 cells were transiently co-transfected with the reporter vector containing the respective 3′-UTR and either miR-218 mimics, miR-520a mimics or the NC. Luciferase activity was assayed at 48 h post-transfection using a Pikkagene Dual Luciferase Reporter Assay system (TOYO B-Net Co., Ltd., Tokyo, Japan).

### Statistical analysis

All experiments were conducted in triplicate. Values are expressed as the mean ± standard deviation. Differences between two groups and in more than two groups were analyzed using Student’s t-test and one-way analysis of variance, respectively. Statistical analyses were performed using SPSS version 16.0 software (SPSS Inc., Chicago, IL, USA). All statistical analyses were two-tailed and P<0.05 was considered to indicate a statistically significant difference between values.

## Results

### miR-218 and miR-520a expression are downregulated in human HCC cell lines

**Figure 1:**
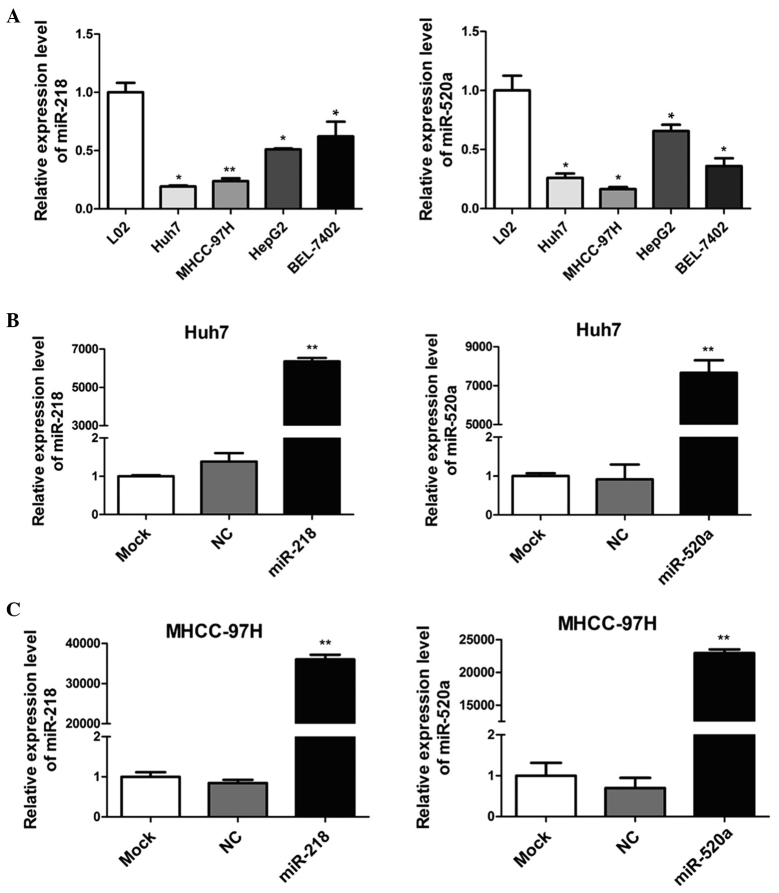
Identification of miR-218 and miR-520a expression in human HCC cell lines. (A) Expression levels of miR-218 and miR-520a were determined by RT-qPCR in the four human HCC cell lines Huh7, MHCC-97H, HepG2 and BEL-7402, as well as the human normal hepatic cell line L02. RT-qPCR was performed to detect the expression levels of miR-218 and miR-520a in (B) Huh7 and (C) MHCC-97H cells transfected with miR-218 or miR-520a. Values are expressed as the mean ± standard deviation (n=3). ^*^P<0.05; ^**^P<0.01, vs. L02 or NC. miR-218, microRNA-218; HCC, hepatocellular carcinoma; RT-qPCR, reverse transcription-quantitative polymerase chain reaction; NC, negative control.

To detect the expression of miR-218 and miR-520a in human HCC cells, RT-qPCR was applied to determine the miRNA expression in four HCC cell lines (Huh7, MHCC-97H, HepG2 and BEL-7402), compared with a human normal hepatic cell line (L02). As demonstrated in Fig. 1A, miR-218 and miR-520a expression levels were significantly downregulated in human HCC cells. In addition, Huh7 and MHCC-97H cells appeared to express lower levels of miR-218 and miR-520a than HepG2 and BEL-7402 cells. These results suggested that miR-218 and miR-520a may serve as suppressors in the development of HCC.

### miR-218 and miR-520a inhibit the proliferation of HCC cells

To investigate the roles of miR-218 and miR-520a in HCC, mimics of miR-218 and miR-520a were transfected into Huh7 and MHCC-97H cells. Fig. 1B demonstrates that the expression levels of miR-218 and miR-520a were significantly upregulated (P<0.01) following transfection, indicating that Huh7 and MHCC-97H cells were effective and adjustable models for the functional study of miR-218 and miR-520a expression.

**Figure 2:**
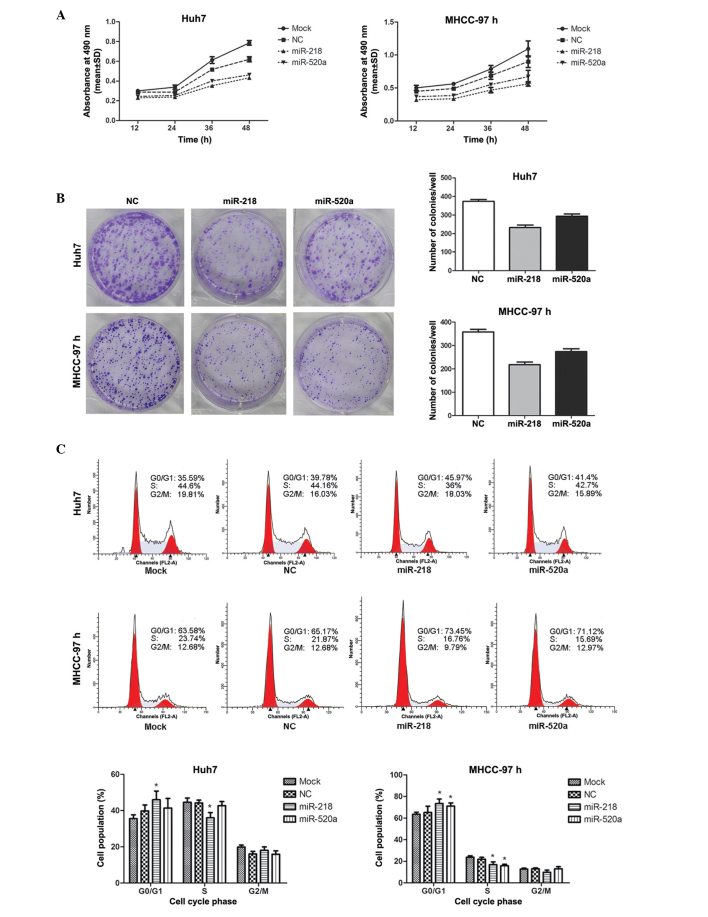
miR-218 and miR-520a inhibit the proliferation of Huh7 and MHCC-97H cells. (A) MTT assay was used to determine the effect of miR-218 and miR-520a on the viability of Huh7 and MHCC-97H cells. Values are presented as the mean ± SD. (B) Colony formation assays of Huh7 and MHCC-97H cells transfected with miR-218 mimics, miR-520a mimics or NC. The bars represent the mean ± SD of three independent experiments. (C) Cell cycle distribution of Huh7 and MHCC-97H cells following transfection of miR-218 mimics, miR-520a mimics or NC. The histograms represent the mean ± SD of three independent experiments. ^*^P<0.05, ^**^P<0.01, vs. NC. miR-218, microRNA-218; SD, standard deviation; NC, negative control.

As presented in Fig. 2A, cell viability was measured by MTT assay. MTT growth curves indicated that the cell proliferative abilities were markedly reduced when miR-218 and miR-520a were overexpressed (P=0.001 for miR-218 in Huh7; P= 0.007 for miR-520a in Huh7; P= 0.002 for miR-218 in MHCC-97; P=0.013 for miR-520a in MHCC-97). Furthermore, the colony formation assay demonstrated that the colony number in cells transfected with miR-218 or miR-520a mimics was significantly lower than that in the NC (P<0.001 for miR-218 in Huh7; P=0.001 for miR-520a in Huh7; P<0.001 for miR-218 in MHCC-97; P=0.001 for miR-520a in MHCC-97), suggesting that the colony-forming abilities of Huh7 and MHCC-97H cells were suppressed by the upregulation of miR-218 or miR-520a (Fig. 2B). These results suggested that miR-218 and miR-520a inhibited the proliferation of the HCC cell lines Huh7 and MHCC-97H.

### miR-218 and miR-520a induce cell cycle arrest in G_0_–G_1_ phase

Flow cytometric analyses demonstrated that the percentages of miR-218-transfected Huh7 and MHCC-97H cells in G_0_-G_1_ phase were 16% (Huh7) and 13% (MHCC-97H) greater than those in the NC group, which is consistent with the reductions of 18% (Huh7) and 23% (MHCC-97H) in the percentage sof cells in S phase. The percentage of miR-520a-transfected MHCC-97H cells in G_0_-G_1_ phase was 9% greater than that in the NC group, which was in parallel with a 27% reduction in S phase, whereas the upregulation of miR-520a exerted no significant effect on the cell cycle distribution of Huh7 cells (Fig. 2C). These data indicated that miR-218 and miR-520a reduced cell proliferation via the induction of cell cycle arrest in G_0_–G_1_ phase.

### miR-218 directly targets the 3′-UTR of E2F2

To further investigate the mechanism of miR-218- and miR-520a-mediated inhibition of cell proliferation, the candidate target genes were assessed using the miRNA target prediction tool miRanda (http://www.microrna.org). Among these genes, including BMI1, MDGA2 and CDK6, E2F2 was predicted as a potential target of miR-218 and miR-520a.

**Figure 3:**
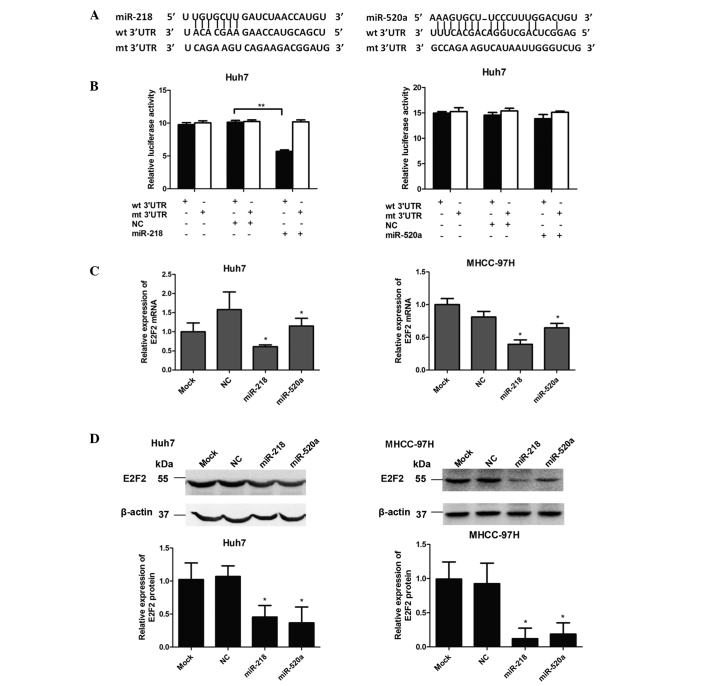
E2F2 is a direct target of miR-218. (A) Predicted binding sites for the seed sequences of miR-218 and miR-520a in the E2F2 3′-UTR and the sites of target mutagenesis are presented. (B) A dual luciferase assay was conducted in order to confirm the direct regulation of miR-218 on the E2F2 3′-UTR. miR-218 and miR-520a regulate E2F2 expression at the (C) mRNA and (D) protein levels in Huh7 and MHCC-97H cells. Values are presented as the mean ± standard deviation (n=3). ^*^P<0.05, ^**^P<0.01, vs. NC. miR-218, microRNA-218; UTR, untranslated region; NC, negative control; Wt, wild-type; mt, mutant.

Dual-luciferase reporter assays were conducted in Huh7 cells to investigate whether E2F2 was a direct target of miR-218 and miR-520a. Fig. 3A presents the potential binding sites of miR-218 and miR-520a in the 3′-UTR of E2F2, with the corresponding sequences of the mutated 3′-UTRs of E2F2 also illustrated. The dual-luciferase reporter assay demonstrated that the luciferase activity was significantly reduced (P<0.01) following co-transfection with miR-218 mimics and the reporter vector bearing the WT 3′-UTR of E2F2. However, no significant difference in luciferase activity was observed when miR-520a mimics were co-transfected with the WT 3′-UTR of E2F2. In addition, the activity in the reporter vector with the mutant 3′-UTR of E2F2 was affected by neither miR-218 nor miR-520a (Fig. 3B). These results suggested that E2F2 is a direct target of miR-218 but not miR-520a.

### miR-218 and miR-520a suppress E2F2 expression

The effects of miR-218 and miR-520a on E2F2 expression were next investigated. As presented in Fig. 3C, compared with the NC group, relative mRNA expression of E2F2 was significantly downregulated (P<0.05) in Huh7 and MHCC-97 cells trans-fected with either miR-218 or miR-520a mimics. Consistent with the results of RT-qPCR, western blot analysis indicated that transfection of Huh7 and MHCC-97 cells with either miR-218 or miR-520a resulted in a significant reduction in E2F2 protein expression (P<0.05; Fig. 3D). Collectively, these results indicated that miR-218 negatively regulated the expression of E2F2 via directly binding to its 3′-UTR, while miR-520a affected E2F2 expression indirectly.

## Discussion

It is widely accepted that miRNAs regulate diverse biological processes, including tumorigenesis. The tumor suppressor miR-218 was reported to be frequently downregulated in various types of cancer (17–25). A previous study has indicated that miR-520a acts as a tumor suppressor in esophageal squamous cell carcinoma (26). In the present study, the expression of miR-218 and miR-520a in HCC cells and the molecular mechanisms by which the miRNAs exert their functions were investigated. First, RT-qPCR was conducted in order to examine the expression levels of miR-218 and miR-520a in four human HCC cell lines and a normal hepatic cell line. The results indicated that the levels of miR-218 and miR-520a were downregulated in cancer cells, which is consistent with the results of previous studies (17–26). In addition, Huh7 and MHCC-97H cells were observed to exhibit low levels of miR-218 and miR-520a expression amongst the four HCC cell lines examined. In accordance with this, the Huh7 and MHCC-97H cell lines were selected for the experiments in the present study. It was hypothesized that miR-218 and miR-520a may serve as tumor suppressors in HCC. The MTT and colony formation assay demonstrated that HCC cells transfected with miR-218 or miR-520a mimics exhibited reduced proliferative abilities compared with those of the control cells. Furthermore, overexpression of miR-218 or miR-520a was observed to induce cell cycle arrest at the G_0_/G_1_ phase checkpoint. Therefore, it was inferred that miR-218 and miR-520a function as tumor suppressors in HCC.

To understand the molecular mechanisms by which miR-218 and miR-520a inhibit cell proliferation in HCC, bioinformatics-based predictions and a dual-luciferase reporter assay were utilized. E2F2 was identified to be a direct target of miR-218 but not miR-520a in HCC. In addition, RT-qPCR and western blot analyses were conducted in order to investigate whether miR-218 and miR-520a may regulate E2F2 expression. The results demonstrated that upregulation of miR-218 or miR-520a downregulated E2F2 mRNA and protein levels. Collectively, it was concluded that miR-218 negatively regulates E2F2 expression via directly binding to its 3′-UTR, whereas miR-520a affects E2F2 expression indirectly.

As critical cell cycle regulators, E2F proteins function downstream of cell cycle signaling cascades and are vital in cell proliferation and growth via the modulation of genes involved in cell cycle progression (27–29). E2F2, a member of the E2F family, activates the transcription of E2F target genes and regulates the G_1_/S-phase transition (28). Previous studies have demonstrated that E2F2 has a strong oncogenic capacity and is able to promote cell-cycle progression (30). Additional previous studies have provided evidence that E2F2 is upregulated in HCC and may be crucial in the promotion of cell proliferation (31,32). In normal cell cycle control, the phosphorylation of cyclin-dependent kinases (CDKs) is essential, which results in phosphorylation of Rb family proteins. Rb subsequently activates E2Fs and allows G_1_/S-phase transition (33). miR-218 has been reported to inhibit CDK4 expression in colon cancer (34). Based on these factors, it is hypothesized that miR-218 regulates E2F2 expression; this occurs not only by directly targeting E2F2 but additionally via the suppression of CDK4; however, further studies are required to validate this hypothesis. Regarding miR-520a, even though E2F2 is not the direct target, the molecular mechanism may be associated with E2F2.

In conclusion, the present study provided evidence that miR-218 and miR-520a are downregulated in HCC, and that these miRNAs act as tumor suppressors to inhibit cell proliferation, partly by regulating E2F2 expression. This suggested that miR-218 and miR-520a may be promising candidates for therapeutic intervention in HCC.

## References

[1] Ambros V (2004). The functions of animal microRNAs. Nature.

[2] Baker AH, van Rooij E (2013). miRNA overexpression induces cardiomyocyte proliferation in vivo. Mol Ther.

[3] Heinzelmann J, Henning B, Sanjmyatav J (2011). Specific miRNA signatures are associated with metastasis and poor prognosis in clear cell renal cell carcinoma. World J Urol.

[4] Zhang JF, Fu WM, He ML (2011). MiRNA-20a promotes osteogenic differentiation of human mesenchymal stem cells by co-regulating BMP signaling. RNA Biol.

[5] Calin GA, Croce CM (2006). MicroRNA signatures in human cancers. Nat Rev Cancer.

[6] Ventura A, Jacks T (2009). MicroRNAs and cancer: short RNAs go a long way. Cell.

[7] Bosch FX, Ribes J, Díaz M, Cléries R (2004). Primary liver cancer: worldwide incidence and trends. Gastroenterology.

[8] Jemal A, Bray F, Center MM, Ferlay J, Ward E, Forman D (2011). Global cancer statistics. CA Cancer J Clin.

[9] El-Serag HB (2011). Hepatocellular carcinoma. N Engl J Med.

[10] Giordano S, Columbano A (2013). MicroRNAs: new tools for diagnosis, prognosis, and therapy in hepatocellular carcinoma?. Hepatology.

[11] Augello C, Vaira V, Caruso L (2012). MicroRNA profiling of hepatocarcinogenesis identifies C19MC cluster as a novel prognostic biomarker in hepatocellular carcinoma. Liver Int.

[12] Garofalo M, Di Leva G, Romano G (2009). miR-221&222 regulate TRAIL resistance and enhance tumorigenicity through PTEN and TIMP3 downregulation. Cancer Cell.

[13] Gramantieri L, Ferracin M, Fornari F (2007). Cyclin G1 is a target of miR-122a, a microRNA frequently down-regulated in human hepatocellular carcinoma. Cancer Res.

[14] Kota J, Chivukula RR, O’Donnell KA (2009). Therapeutic microRNA delivery suppresses tumorigenesis in a murine liver cancer model. Cell.

[15] Meng F, Henson R, Wehbe-Janek H, Ghoshal K, Jacob ST, Patel T (2007). MicroRNA-21 regulates expression of the PTEN tumor suppressor gene in human hepatocellular cancer. Gastroenterology.

[16] Zhu Y, Lu Y, Zhang Q (2012). MicroRNA-26a/b and their host genes cooperate to inhibit the G1/S transition by activating the pRb protein. Nucleic Acids Res.

[17] Alajez NM, Lenarduzzi M, Ito E (2011). MiR-218 suppresses nasopharyngeal cancer progression through downregulation of survivin and the SLIT2-ROBO1 pathway. Cancer Res.

[18] Gao CP, Zhang Z, Liu W, Xiao S, Gu W, Lu H (2010). Reduced microRNA-218 expression is associated with high nuclear factor kappa B activation in gastric cancer. Cancer.

[19] Li Q, Zhu F, Chen P (2012). miR-7 and miR-218 epigenetically control tumor suppressor genes RASSF1A and Claudin-6 by targeting HoxB3 in breast cancer. Biochem Biophys Res Commun.

[20] Liu Y, Yan W, Zhang W (2012). MiR-218 reverses high invasiveness of glioblastoma cells by targeting the oncogenic transcription factor LEF1. Oncol Rep.

[21] Martinez I, Gardiner AS, Board KF, Monzon FA, Edwards RP, Khan SA (2008). Human papillomavirus type 16 reduces the expression of microRNA-218 in cervical carcinoma cells. Oncogene.

[22] Uesugi A, Kozaki K, Tsuruta T (2011). The tumor suppressive microRNA miR-218 targets the mTOR component rictor and inhibits AKT phosphorylation in oral cancer. Cancer Research.

[23] White NMA, Bao TT, Grigull J (2011). miRNA profiling for clear cell renal cell carcinoma: biomarker discovery and identification of potential controls and consequences of miRNA dysregulation. J Urol.

[24] Wu DW, Cheng YW, Wang J, Chen CY, Lee H (2010). Paxillin predicts survival and relapse in non-small cell lung cancer by microRNA-218 targeting. Cancer Res.

[25] Li C, Tu K, Zheng X (2013). MicroRNA-218 expression and its role in hepatocellular carcinoma. Nan Fang Yi Ke Da Xue Xue Bao.

[26] Ye W, Yao Q, Zhang M, Wen Q, Wang J (2014). miR-520a regulates ErbB4 expression and suppresses proliferation and invasion of esophageal squamous cell carcinoma. Nan Fang Yi Ke Da Xue Xue Bao.

[27] Attwooll C, Lazzerini Denchi E, Helin K (2004). The E2F family: specific functions and overlapping interests. EMBO J.

[28] Dimova DK, Dyson NJ (2005). The E2F transcriptional network: old acquaintances with new faces. Oncogene.

[29] Ren B, Cam H, Takahashi Y (2002). E2F integrates cell cycle progression with DNA repair, replication, and G(2)/M checkpoints. Genes Dev.

[30] Chen C, Wells AD (2007). Comparative analysis of E2F family member oncogenic activity. PLoS ONE.

[31] Santoni-Rugiu E, Jensen MR, Thorgeirsson SS (1998). Disruption of the pRb/E2F pathway and inhibition of apoptosis are major oncogenic events in liver constitutively expressing c-myc and transforming growth factor alpha. Cancer Res.

[32] Zhan L, Huang C, Meng XM (2014). Promising roles of mammalian E2Fs in hepatocellular carcinoma. Cell Signal.

[33] Dong Q, Meng P, Wang T (2010). MicroRNA let-7a inhibits proliferation of human prostate cancer cells in vitro and in vivo by targeting E2F2 and CCND2. PLoS ONE.

[34] He XQ, Dong YJ, Wu CW (2012). MicroRNA-218 inhibits cell cycle progression and promotes apoptosis in colon cancer by downregulating BMI1 polycomb ring finger oncogene. Mol Med.

